# Tuina for children with upper respiratory tract infections: A protocol for a systematic review

**DOI:** 10.1097/MD.0000000000016443

**Published:** 2019-07-12

**Authors:** Jiayuan Zhang, Yunhui Chen, Liu Cao, Renyan Zhang, Renyuan Ren, Qi Zhang

**Affiliations:** a Chengdu University of Traditional Chinese Medicine, No. 37 Shierqiao Road, Jinniu District; b Sichuan Integrative Medicine Hospital, Wuhou District, Chengdu, Sichuan, China.

**Keywords:** children, protocol, systematic review, Tuina, upper respiratory tract infections

## Abstract

**Background:**

Upper respiratory tract infections (URTIs) is a common disease in children, which is also known as the common cold. Pediatric Tuina is a common treatment that Traditional Chinese Medicine (TCM) doctors commonly use for URTIs. However, there has no relevant systematic review studied on its effects and safety been reported. We plan to perform a systematically reviewing of all the clinical evidence on the effectiveness and safety of Tuina for URTIs in children.

**Methods:**

We will conduct the literature searching in the following electronic databases: Pubmed, Embase, Cochrane Library, Web of science, Chinese National Knowledge Infrastructure (CNKI), VIP, Wanfang, China Biomedical Literature Database (CBM), Chinese Clinical Trial Registry System. The time limit for retrieving studies is from establishment to July 2019 for each database. All published randomized controlled trials (RTCs) related to this review will be included. Review Manager (V.5.3.5) will be implemented for the assessment of bias risk and data analyses. Subgroup analysis and sensitivity analysis will be performed based on the conditions of included data.

**Results:**

A high-quality synthesis of current evidence of Tuina for children with URTIs will be provided in this study.

**Conclusion:**

This systematic review will provide evidence of whether Tuina is an effective intervention for children with URTIs.

**PROSPERO registration number:**

CRD42019126963.

## 1 Introduction

Upper respiratory tract infections (URTIs) is a common disease in children whose symptoms include cough, sore throat, nasal stuffiness and discharge, sometimes accompanied with fever, also known as the common cold.^[1–3]^ URTIs is the major cause of pediatric outpatient visits; every child has an average of 6 colds per year.^[2,4]^ Although URTIs is mostly self-limiting, the possibility of developing to lower respiratory tract infections, tympanitis, asthma and febrile seizures is relatively high for children. As the URTIs is mainly caused by a multitude of different virus types with varying pathogenetic mechanisms,^[5,6]^ the effectiveness and safety of current symptomatic interventions are uncertain for children. Widely use of antibiotics in URTIs is highly controversial for unsatisfactory effect on viruses.^[7–11]^ And inappropriate antibiotic prescriptions in children are more likely to lead to drug resistance, and may also increase the risk of diseases such as antibiotic-associated diarrhea, increase diabetes, autoimmune diseases, asthma, obesity, and so on.^[9,12–14]^

Tuina is a physical therapy derived from Chinese medicine, using a series of manipulation on the human body surface to treat the disease. Pediatric Tuina is more easily accepted by children than drugs, especially for younger children who have difficulties taking medication. Pediatric Tuina is gradually widely used in China. The specific acupoint theory and operation methods are employed in pediatric Tuina to treat the common disease of children such as respiratory infections, anorexia, diarrhea, cerebral palsy, dysplasia, and so on.^[15–19]^ Traditional Chinese medicine (TCM) doctors often apply pediatric Tuina to relieve the symptoms of acute URTIs and reduce the incidence of repeated respiratory infections. Studies have shown that Tuina can regulate the immune system function.^[20,21]^ However, the current clinical efficacy lacks evidence-based medical system evaluation. Therefore, this study conducted a systematic review to objectively evaluate the efficacy of Tuina treatment for respiratory tract infections in children.

## 2 Method

### 2.1 Selection criteria

#### 2.1.1 Types of studies

We will include the RCTs that involved Tuina therapy for URTIs in children in the treatment group, and its control group used other kinds of treatment without Tuina therapy. The literature of animal research, case report, review, and meta-analysis will be excluded.

#### 2.1.2 Types of patients

Children (<12 years old) diagnosed with URTIs will be included in the study. There is no limitation on the gender, nation, and duration of the disease. Patients with severe medical conditions will be excluded.

#### 2.1.3 Types of interventions

RCT using Tuina manipulation or Tuina with drugs, other treatments or no treatment(waiting-listed) for comparison will be included. RCT with comparison involved Tuina manipulation will be excluded.

#### 2.1.4 Types of outcomes

The main outcome of this review is the effective rate after treatments. Additional outcomes are reduced symptom scores, recurrence rate and adverse events including hematoma.

### 2.2 Search strategy

Studies published on the Pubmed, Embase, Cochrane Library, Web of science, Chinese National Knowledge Infrastructure (CNKI), VIP, Wanfang, China Biomedical Literature Database (CBM), Chinese Clinical Trial Registry System will be searched since the establishment to August 2019.

The search keywords used include the following: “upper respiratory infections,” “upper respiratory tract infections,” “common cold,” “acute Coryza,” “Massage,” “Tuina,” “manipulation,” “Massotherapy,” “Child,” “Children,” “Infant,” “pediatric,” and “random” and there will be no restriction on language. More details will be updated on the “PROSPERO” website; the registration number is CRD42019126963. This study is an evidence-based medical research, so the ethical approval is not necessary.

### 2.3 Data collection and analysis

#### 2.3.1 Selection of studies

**Figure 1:**
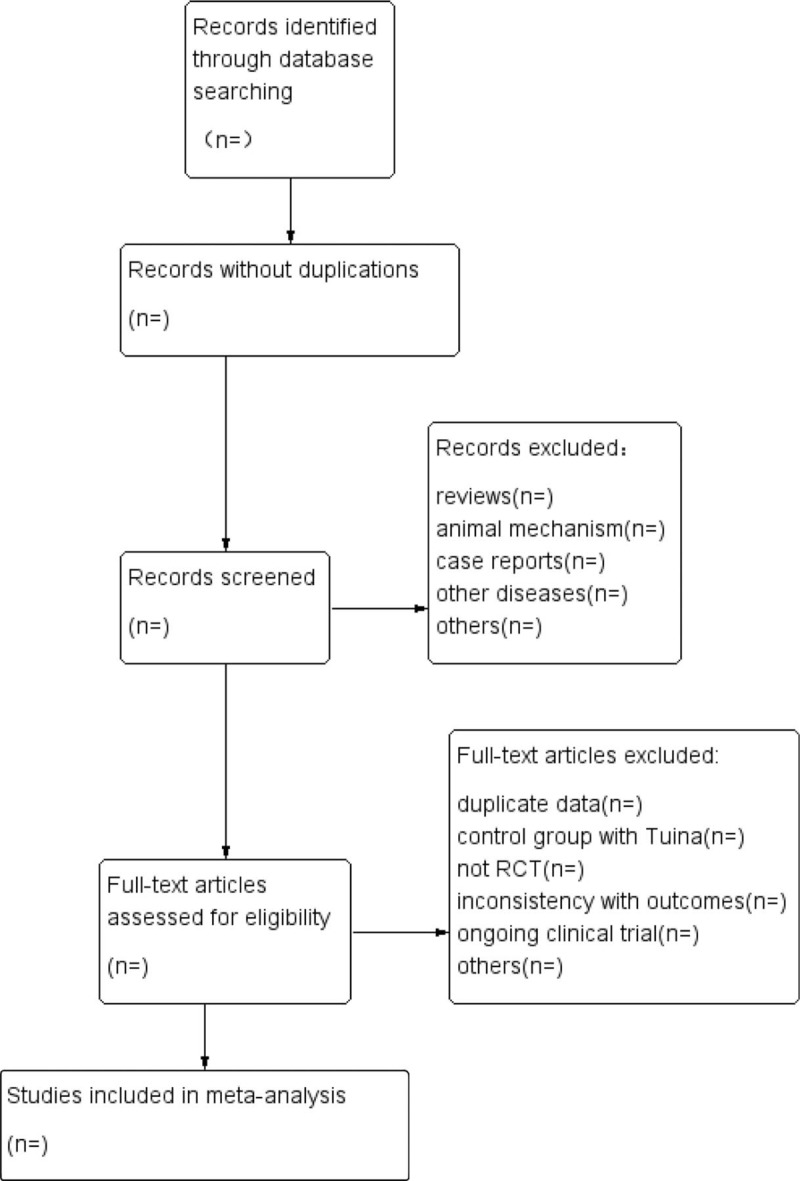
Flow of literature screening.

Literature screening, study selection, and data extraction were performed by 2 reviewers (Jiayuan Zhang, Liu Cao). The literature searched from the electronic database will be imported EndnoteX9 for further screening of title and abstract, the duplications, and studies not meet the inclusion criteria will be excluded. After reading the full text of the remained literature, discussing within the group, the final included studies will be determined. We will try to contact the corresponding author when full text unavailable. Disagreements were solved by discussion or consulting a third party arbitrator until a consensus was achieved. The entire process of study selection is performed in the PRISMA flow diagram (Fig. 1).

#### 2.3.2 Data extraction

The 2 reviewers will independently extract data by a standard form which includes title (serial number, name of first author, journal source, and published date), participants (age, gender, and number), interventions (method, time, and cycle), main outcomes, additional outcomes, and adverse events. If the data provided in the research are unclear, missing or difficult to extract reliably, the corresponding author will be contacted for clarification.

#### 2.3.3 Assessment of risk of bias in included studies

The quality assessment for each RCT will be conducted independently by 2 reviewers using the Cochrane Collaboration Risk of Bias Tool checklist. Random sequence generation, allocation concealment, blinding of participants and personnel, blinding of outcome assessment, incomplete outcome data, selective reporting, and other bias will be assessed as low risk, high risk, or ambiguous risk in each RCT. Third-party experts will be consulted during any disagreement.

#### 2.3.4 Measures of treatment effect

The treatment effect will be analyzed by 2 independent reviewers with Review Manager 5.3 software provided by the Cochrane Collaboration. Risk ratio (RR) with 95% confidence interval (CI) will be adopted for the dichotomous data (e.g., effective rate). For continuous data (e.g., recurrence rate), the mean difference (MD) with 95% CI will be adopted.

#### 2.3.5 Dealing with missing data

The corresponding authors of the included studies with missing data will be contacted by available contact ways. If data are still unattainable, the study will be excluded in the data analysis.

#### 2.3.6 Assessment of heterogeneity

Heterogeneity of the data will be assessed by *Q* test and *I*^2^ statistic. The heterogeneity will be deemed as low when *I*^2^ < 50%, moderate (50%–75%), high (*I*^2^ > 75%). The cause of the heterogeneity will be analyzed and a subgroup analysis will be performed.

#### 2.3.7 Assessment of reporting bias

A funnel plot will be conducted to assess publication bias when the number of sufficient studies included for data analysis is >10.

#### 2.3.8 Data synthesis

We will perform the systematic review of the literature with meta-analysis based on measurement methods, intervention methods, and the length of treatment and heterogeneity levels, and so on. Fixed-effect model will be applied when heterogeneity is low. Random-effects model will be applied when heterogeneity is moderate. Although the heterogeneity is significantly high, subgroup analysis or descriptive analysis will be performed.

#### 2.3.9 Subgroup analysis

Subgroup analysis will be performed to assess the high heterogeneity of included studies. We will conduct subgroup analysis based on the data, such as disease duration, intervention time, different acupuncture points for Tuina, and so on.

#### 2.3.10 Sensitivity analysis

After the quality assessment of the included literature, if there are possible low-quality studies, sensitivity analysis will be required. We will observe fluctuation of termination by changing the genre of research (incorporating or excluding a particular study) and reanalysis of simulated missing data.

## 3 Discussion

URTIs is very common during childhood. Symptomatic treatment in the acute phase does not reduce recurrence, and the use of antibiotics is not necessary in many cases. As a noninvasive external physiotherapy that China has used for thousands of years, TCM doctors believe that Tuina is effective and environmental-friendly with low cost, and especially suitable for children with fever and history of repeated respiratory infections.^[22]^ However, up to now, there has no relevant systematic review been reported. This review aims to objectively evaluate the effectiveness and safety of pediatric Tuina in the treatment of URTIs in children based on evidence-based medicine, and whether it can effectively relieve acute URTIs and reduce the incidence of repeated URTIs. Due to the particularity of Tuina technology (TCM technology), it is expected that the RCTs retrieved in this review are mostly conducted in China, and the differences in specific treatment regimens and methodological quality in each trial can lead to significant heterogeneity.

## Author contributions

**Conceptualization:** Jiayuan Zhang, Yunhui Chen, Qi Zhang.

**Data curation:** Yunhui Chen, Liu Cao.

**Formal analysis:** Jiayuan Zhang, Yunhui Chen, Liu Cao.

**Funding acquisition:** Yunhui Chen.

**Investigation:** Renyan Zhang, Renyuan Ren.

**Methodology:** Jiayuan Zhang, Liu Cao, Renyan Zhang, Renyuan Ren.

**Project administration:** Renyan Zhang, Qi Zhang.

**Resources:** Liu Cao.

**Software:** Jiayuan Zhang.

**Supervision:** Renyan Zhang.

**Visualization:** Renyuan Ren.

**Writing – original draft:** Jiayuan Zhang, Liu Cao.

**Writing – review and editing:** Yunhui Chen, Qi Zhang.

## References

[1] Heikkinen T Jarvinen A The common cold. Lancet 2003;361:51–9. 12517470 10.1016/S0140-6736(03)12162-9 PMC7112468

[2] van Driel ML Scheire S Deckx L What treatments are effective for common cold in adults and children? BMJ 2018;363 (k3786):10.1136/bmj.k3786 30305295

[3] Eccles R Understanding the symptoms of the common cold and influenza. Lancet Infect Dis 2005;5:718–25. 16253889 10.1016/S1473-3099(05)70270-X PMC7185637

[4] Allan GM Arroll B Prevention and treatment of the common cold: making sense of the evidence. Can Med Assoc J 2014;186:190–9. 24468694 10.1503/cmaj.121442 PMC3928210

[5] Gwaltney JJ Wiesinger BA Patrie JT Acute community-acquired bacterial sinusitis: the value of antimicrobial treatment and the natural history. Clin Infect Dis 2004;38:227–33. 14699455 10.1086/380641

[6] Makela MJ Puhakka T Ruuskanen O Viruses and bacteria in the etiology of the common cold. J Clin Microbiol 1998;36:539–42. 9466772 10.1128/jcm.36.2.539-542.1998 PMC104573

[7] Andrade JV Vasconcelos P Campos J Antibiotic prescribing in ambulatory care of pediatric patients with respiratory infections. Acta Medica Port 2019;32:101–10. 10.20344/amp.11111 30896390

[8] van de Voort EMF Mintegi S Gervaix A Antibiotic use in febrile children presenting to the emergency department: a systematic review. Front Pediatr 2018;6:10.3389/fped.2018.00260 PMC6186802 30349814

[9] Yonts AB Kronman MP Hamdy RF The burden and impact of antibiotic prescribing in ambulatory pediatrics. Curr Prob Pediatr Adolesc Health Care 2018;48:272–88. 10.1016/j.cppeds.2018.09.002 30337150

[10] Mangione-Smith R Zhou C Robinson JD Communication practices and antibiotic use for acute respiratory tract infections in children. Ann Fam Med 2015;13:221–7. 25964399 10.1370/afm.1785 PMC4427416

[11] Hersh AL Shapiro DJ Pavia AT Antibiotic prescribing in ambulatory pediatrics in the United States. Pediatrics 2011;128:1053–61. 22065263 10.1542/peds.2011-1337

[12] Kronman MP Zaoutis TE Haynes K Antibiotic exposure and IBD development among children: a population-based cohort study. Pediatrics 2012;130:E794–803. 23008454 10.1542/peds.2011-3886 PMC4074626

[13] Stevens V Dumyati G Fine LS Cumulative antibiotic exposures over time and the risk of clostridium difficile infection. Clin Infect Dis 2011;53:42–8. 21653301 10.1093/cid/cir301

[14] Shao X Ding X Wang B Antibiotic exposure in early life increases risk of childhood obesity: a systematic review and meta-analysis. Front Endocrinol 2017;8:10.3389/fendo.2017.00170 PMC5517403 28775712

[15] Peng R Wang R Cheng N A systematic review and GRADE evaluation of randomized controlled trials of prolonged and chronic diarrhea in children under 12 years old with pediatric Tuina. J Tradit Chin Med 2018;59:1747–52.

[16] Su X Zhong Z Qiao X Clinical study on Tuina treating acute upper respiratory tract infections in infants. J Basic Chin Med 2017;23:1750–1.

[17] Yang C Lu M Yu T Systematic review and meta-analysis of randomized controlled trials of tuina in treatment of infantile anorexia. Chin Arch Tradit Chin Med 2017;35:1161–6.

[18] Luo G Liu Z Therapeutic effect of TCM massage on infantile cerebral palsy. Chi J Rehab Theory Pract 2012;18:654–7.

[19] Wang C Wang C Feng J Clinical observation on the value of early intervention of traditional Chinese medical massage on high-risk infants. Chin J Child Health Care 2018;26:459–61.

[20] Xia T Fu J Tang H Clinical observation on the effect of tuina in prevention and treatment of recurrent respiratory infection induced by pediatric lung Qi deficiency. J Nanjing Univ Tradit Chin Med 2018;34:273–6.

[21] Li Q Tian F Cui J Effect of massage on IL-17, IL-33 and IL-6 of pediatric asthma in different time. MaternChild Health Care China 2014;29:530–2.

[22] Wang L Wang S Han X Guidelines for TCM diagnosis and treatment of repeated respiratory infections in children. J Pediatr Tradit Chin Med 2008;6:3–4.

